# Supplementary material for: Role of inter-hemispheric connections in functional brain networks
Source: Sci Rep. 2018 Jul 6;8:10246. doi: 10.1038/s41598-018-28467-x (PMC6035280; doi:10.1038/s41598-018-28467-x)
Supplement: Supplementary file 1 — Supplementary Information [file 41598_2018_28467_MOESM1_ESM.pdf]

Supplementary Information

Role of inter-hemispheric connections in functional brain networks

J. H. Martínez*, J. M. Buldú, D. Papo, F. De Vico Fallani, M. Chavez

The following results complement the information in the main text. Here we condensed the results obtained from frequency bands θ, α, β and γ for both EC and EO conditions.

Functional Balance in fully connected Network-of-Networks

In Supplementary Fig. S1, we observe the positive trends between local and global centralities for the averaged connectivity matrices associated to each band and each condition (EC and EO). This positive trend suggests a sort of centrality balance when the full connected networks are taken into account regardless bands and conditions. All bands and conditions behave in a similar way and Table S1 summarizes the goodness of the linear fits in the population.

	Panel **A**			Panel **B**			Panel **C**			Panel **D**		
Band	m	b	r^2	m	b	r^2	m	b	r^2	m	b	r^2
θ	0.61	0.02	0.94	0.55	0.03	0.79	0.48	0.04	0.86	0.82	-0.02	0.94
α	0.57	0.03	0.78	0.73	-0.01	0.86	0.70	0.0	0.96	0.73	-0.004	0.97
β	0.74	0.01	0.95	0.63	-0.002	0.80	0.57	0.02	0.91	0.74	-0.002	0.86
γ	0.47	0.05	0.69	0.62	0.01	0.78	0.52	0.04	0.49	0.49	-0.04	0.72

Table S1: Numerical results from linear fits in both conditions and all bands. Here m and b are the statistics of the model $y = mx + b$ with r^2 as coefficient of determination who measures how well the model predict the data ($0 \leq r^2 \leq 1$). Each macro columns represents results for each panel of Fig.S1.

*Corresponding author: johemart@gmail.com

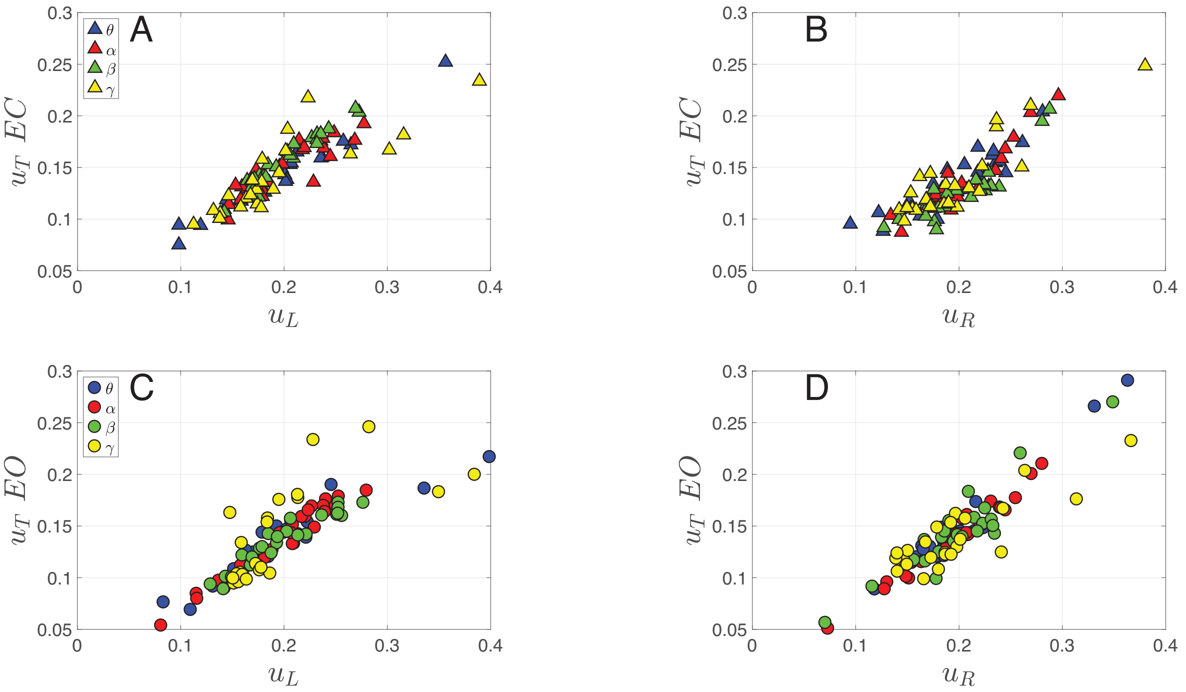

Figure S1: **Global and local centrality for all bands in EC and EO conditions.** Global centrality u_T is obtained from the complete matrix T, when all functional connections between hemispheres are maintained (horizontal axes); while local centrality $u_{L,R}$ is extracted from the hemisphere matrices L and R when hemispheres are disconnected (vertical axes). Frequency bands θ (blue), α (red), β (green), γ (yellow) are drew in triangle and circle markers for EC and EO conditions, respectively. Upper panel shows the EC condition for left (**A**) and right (**B**) hemispheres. Bottom panel: for left (C) and right (D) hemispheres in the EO condition. Table S1 for statistics of linear fits.

We also compute the centrality contrast and competition parameter for all bands in both conditions. Figure S2 shows the violin plots and means of these global features. We do not find statistical differences between each mean values and a zero mean distribution by means of Mann-Whitney U Tests. Summarized results are shown in Table S2. Slight differences in the hemispherical importance are observed when both the EC and EO conditions are compared. We observe positive values of centrality contrast in the left hemisphere during the EC condition for all bands. On the contrary, we report negative values of centrality contrast when individuals open their eyes, which indicates a slight imbalance of centrality between the hemispheres in this condition (see Fig. S2**A** and second column of Table S2).

Interestingly, results in Fig. S2**B** and Table S2) show that $\langle \Omega_L \rangle$ for α, β and γ are slightly negative. Since the competition parameter is defined taking the left hemisphere as reference, negative values of $\langle \Omega_L \rangle$ are consequence of an inter-hemispheric link distribution that slightly benefits the right hemisphere.

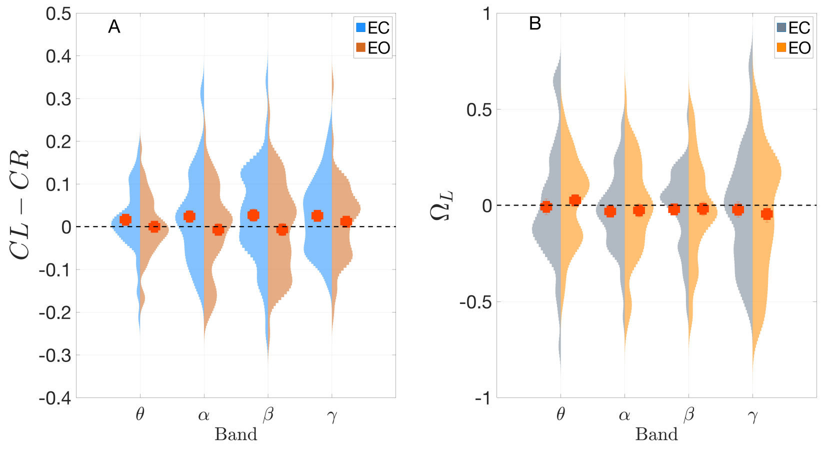

Figure S2: Centrality contrast and Competition parameter. EC conditions (blue for centrality contrast and gray for Ω) and EO (orange for centrality contrast and yellow for Ω)) and four frequency bands: θ, α, β, γ. **A**. Average values of centrality contrast ($\langle C_L - C_R \rangle$) over 54 subjects are indicated by the red circles. **B**. Average of the Competition Parameter $\langle \Omega_L \rangle$ for all subjects.

	$\langle \mathbf{C_L} - \mathbf{C_R} \rangle$		$\langle \Omega_L \rangle$	
Band	EC	EO	EC	EO
θ	0.016	0.0	−0.008	0.025
α	0.023	−0.007	−0.032	−0.026
β	0.026	−0.007	−0.021	−0.018
γ	0.025	0.011	−0.022	−0.044

Table S2: **Mean centralities contrast and competition parameter** Ω_L. Numerical results obtained from Fig. S2 in both conditions.

Competition parameter: An example

For illustrative purposes, Fig. S3 exemplifies the rules explained in the Methods Section about the role of the connector nodes of each hemisphere. These connections lead to the best/worst centrality distribution for the average subject in alpha band. We show the configuration for inter-hemispheric links in three different cases for α-EC. The configuration that makes L to acquire the highest centrality (Fig. S3**A**), relies on the peripheral-peripheral (PP) strategy. The real distribution of centrality (Fig. S3**B**) may use a mixture of CC (central-central), PP, CP and PC strategies. The configuration that allows R hemisphere to get the highest centrality respect the real configuration (Fig. S3**C**) is based on the CC strategy. According to values in each strategy: PP leads to $C^L_{max} \approx 0.7$, the actual configuration gives $C^L = 0.49$ and CC grants $C^R_{max} \approx 0.8$. We obtain $\Omega_L = -0.006$, which reveals the real inter-hemispheric connectivity pattern as promoter of the centrality balance between both hemispheres.

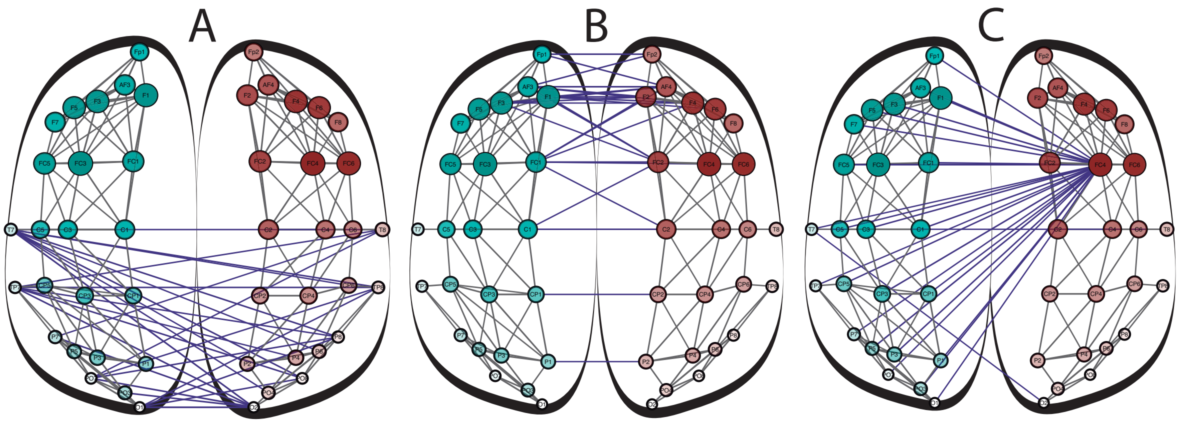

Figure S3: **Example of reshuffling inter-hemispherical links**. Three different configurations of the inter-hemispherical links. Intra-hemispherical connections are colored in grey, while blue is used for the inter-hemispherical links. Nodes' sizes and transparency are proportional to their local eigenvector centrality before connecting both hemispheres. The average α-EC network has an arbitrary threshold that maintains the 16% of the stronger links just for a better visualization purpose. **A**. Peripheral nodes are connected (PP strategy), resulting in optimal strategy for increasing left hemisphere's centrality. **B**. Actual distribution of inter-hemispheric connections, which leads to a balance of the centrality distribution. **C**. Right hemisphere's optimal strategy is obtained by connecting central nodes (CC strategy).

Evolution of the left hemisphere centrality

Figure S4 shows the evolution of C_L (note that $C_R = 1 - C_L$) respect to the inter-hemispheric links. We distinguish between left- and right- dominant individuals based on the eigenvalue. The process of adding inter-hemispheric links shows a clear tendency: the hemisphere that initially has the "strongest" network (i.e., the higher λ_1) acquires a high amount of centrality when the number of inter-hemispherical links is low, but its centrality diminishes as the number of inter-links is increased. "Weak" hemispheres behave just in the opposite way.

We compute the largest eigenvalue λ_1 of L and R and call the "strong" ("weak") hemisphere that with the highest (lowest) λ_1. We also distinguish between groups of people that are *left-dominant* when the eigenvalue λ_1 of L is higher than λ_1 of R, or *right-dominant* in the opposite case. Tab.S3 summarizes the percentage of each type of dominance according to the condition and frequency band.

	Left-dominant population (%)			
Condition	θ	α	β	γ
EC	59.2	57.4	55.5	53.7
EO	37.0	50.0	50.0	55.0

Table S3: Hemisphere dominance is defined according to the largest eigenvalue λ_1 of the hemispheric connectivity matrix: the hemisphere with the highest λ_1 is the one that dominates over the other.

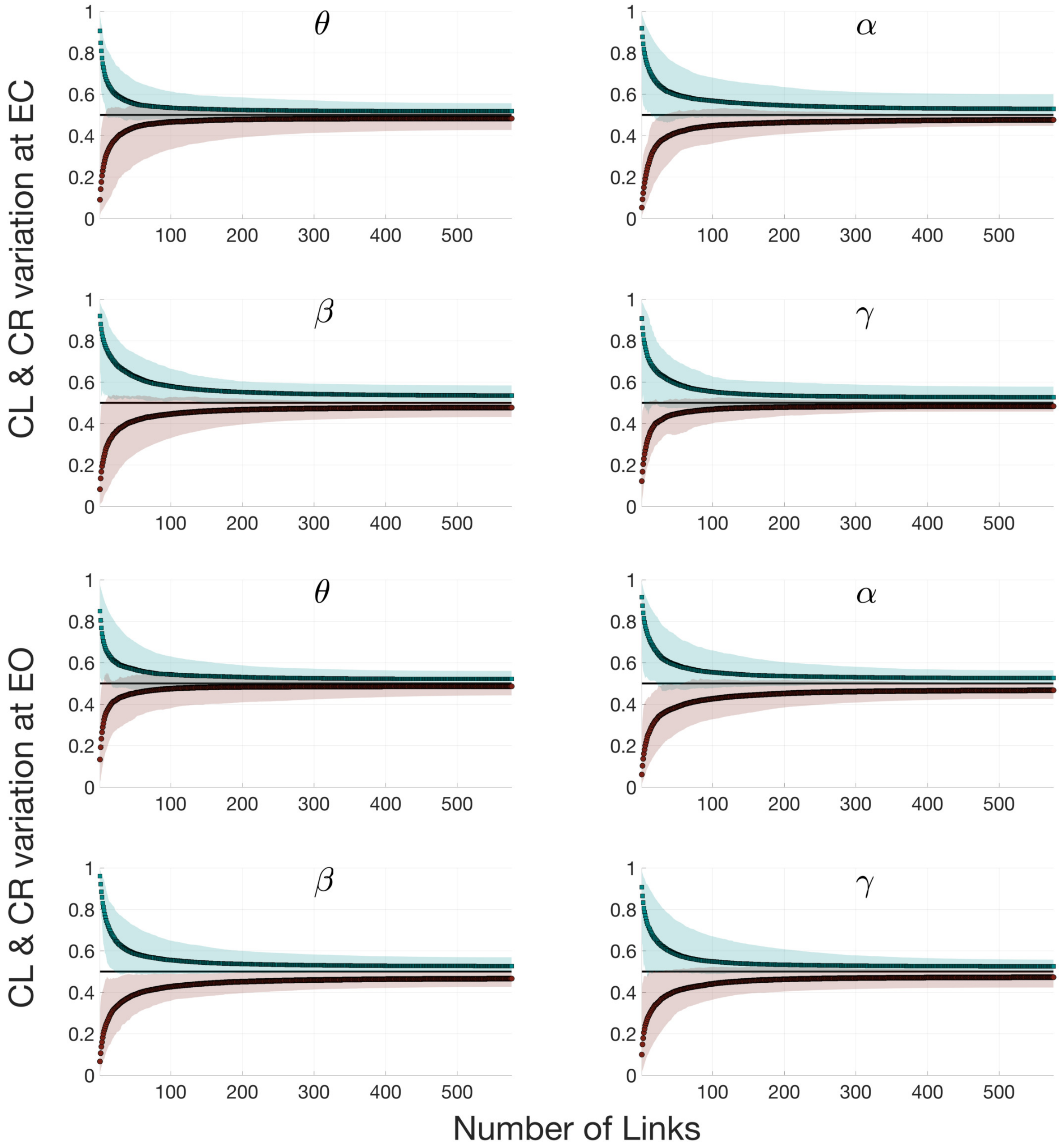

Figure S4: Hemispherical centrality C_L vs. the number of inter-hemispherical links for all subjects. Each subplot shows a different combination of a condition (EC or EO) and frequency band (θ, α, β and γ). Colors indicate whether the dominant hemisphere is the left (green) or the right (red). In each subplot, the region inside each curve includes the fifth and the 95th percentiles of C_L and C_R for all 54 subjects, with the average value plotted in dashed lines.

Robustness and resilience of all bands

We report the results of local impact vs local contribution in the three stages for all bands in both conditions. Tables with the slopes of linear fits between the local impact and local contribution are presented in Table S4 and S5. In Fig. S5 network damage is in the vertical axes and local contribution is in horizontal axes. The values of the three stages are shown for all bands and conditions.

	Stage 3		Stage 2		Stage 1	
Band	m_{EC_L}	m_{EO_L}	m_{EC_L}	m_{EO_L}	m_{EC_L}	m_{EO_L}
θ	−3.784	−4.296	−1.238	−1.357	−0.479	−0.537
α	−3.629	−3.466	−1.541	−1.021	−0.421	−0.465
β	−3.107	−2.416	−1.262	−1.082	−0.599	−0.631
γ	−4.034	−4.098	−1.857	−2.013	−0.686	−0.748

Table S4: **Correlation between the local impact and the local contribution (left hemisphere)**. Slopes of linear fits of $\langle l^L_{imp} \rangle$ vs. $\langle l^L_c \rangle$ for all bands, conditions and stages associated to Fig. S5 **A** y **C**. Note the increase of the slope from Stage 1 to Stage 3 as that described in the main text of the manuscript.

	Stage 3		Stage 2		Stage 1	
Band	m_{EC_R}	m_{EO_R}	m_{EC_R}	m_{EO_R}	m_{EC_R}	m_{EO_R}
θ	−3.942	−4.660	−1.759	−1.070	−0.623	−0.441
α	−4.322	−2.788	−2.000	−1.222	−0.608	−0.466
β	−3.118	−1.430	−1.653	−1.064	−0.532	−0.586
γ	−4.480	−3.534	−2.462	−2.240	−0.833	−0.933

Table S5: **Correlation between the local impact and the local contribution (right hemisphere)**. Slopes of the linear fits of $\langle l^R_{imp} \rangle$ vs $\langle l^R_c \rangle$ for all bands, conditions and stages associated to Fig. S5 **C** y **D**. Note the increase of the slope from Stage 1 to Stage 3 as that described in the main text of the manuscript.

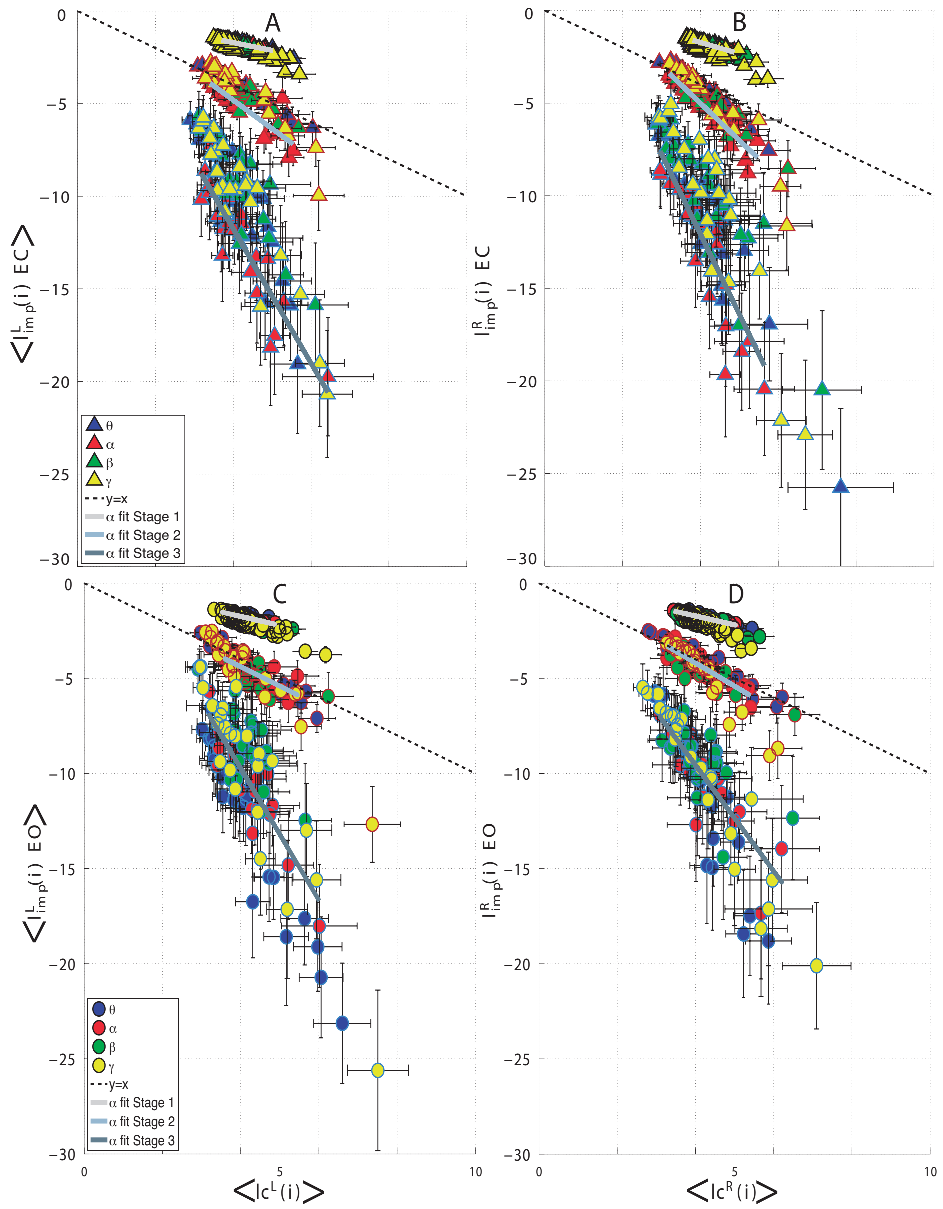

Figure S5: **Local impact $\langle l^L_{imp}(i)\rangle$ vs. local contribution $\langle lc^L(i)\rangle$ during a failure of node** i. In all plots, points (i.e., triangles/circles) correspond to single-node failures (i.e., a single node is removed from the functional network). Triangles (circles) of the Upper (bottom) plots refer to the EC (EO) condition. Left (right) panel refers to the $L(R)$ hemisphere. In each subplot three groups can be identified: (1) Stage 1 corresponds to the upper cloud of nodes, (2) Stage 2 to the middlle cloud and (3) Stage 3 to the bottom group of nodes. The dashed line corresponds to $\langle l^L_{imp}(i)\rangle = \langle lc^L(i)\rangle$. For each stage, a solid line allows to better follow the linear correlation between the local contribution and the local impact.

Figure S6 shows the topological distributions of averaged local impact nodes' positions ate three different stages. In this case, we focus only in α band.

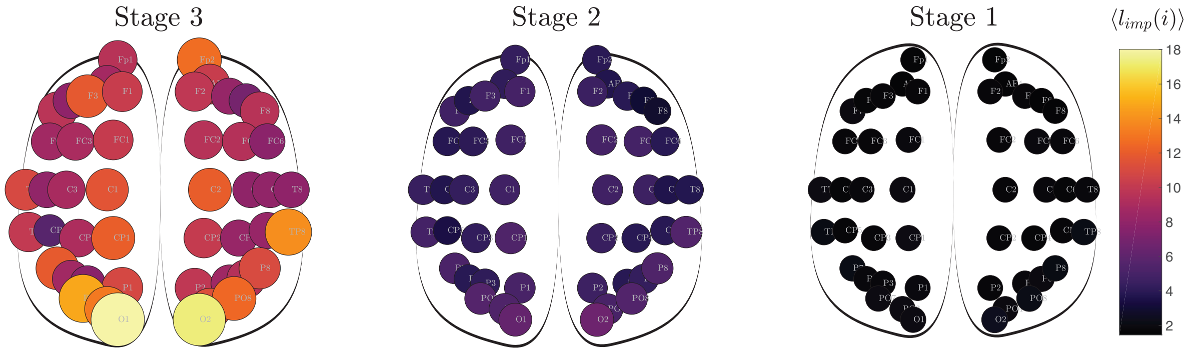

Figure S6: **Local impact in the α band for EO condition**. Average local impacts in α band and EO condition. The radius of each node is proportional to the average local impact. Note how the local impact of the nodes increases as we move from Stage 1 to Stage 3 indicating the occipital lobe as the most vulnerable region.

Local impact on centrality, clustering and shortest path

Figures S7 and Fig. S8 show the behaviour of local impact on centrality, clustering ($\langle l_{imp_c}(i) \rangle$) and shortest path ($\langle l_{imp_d}(i) \rangle$) respect to the local contribution $\langle l_c(i) \rangle$ for all bands and both conditions. In both plots each condition, EC and EO, is represented with triangles and circles, respectively. Frequency bands are also differentiated: θ (blue), α (red), β (green) and γ (yellow). In contrast to the local impact on centrality, clustering and shortest path do not show important changes, no matter the stage considered.

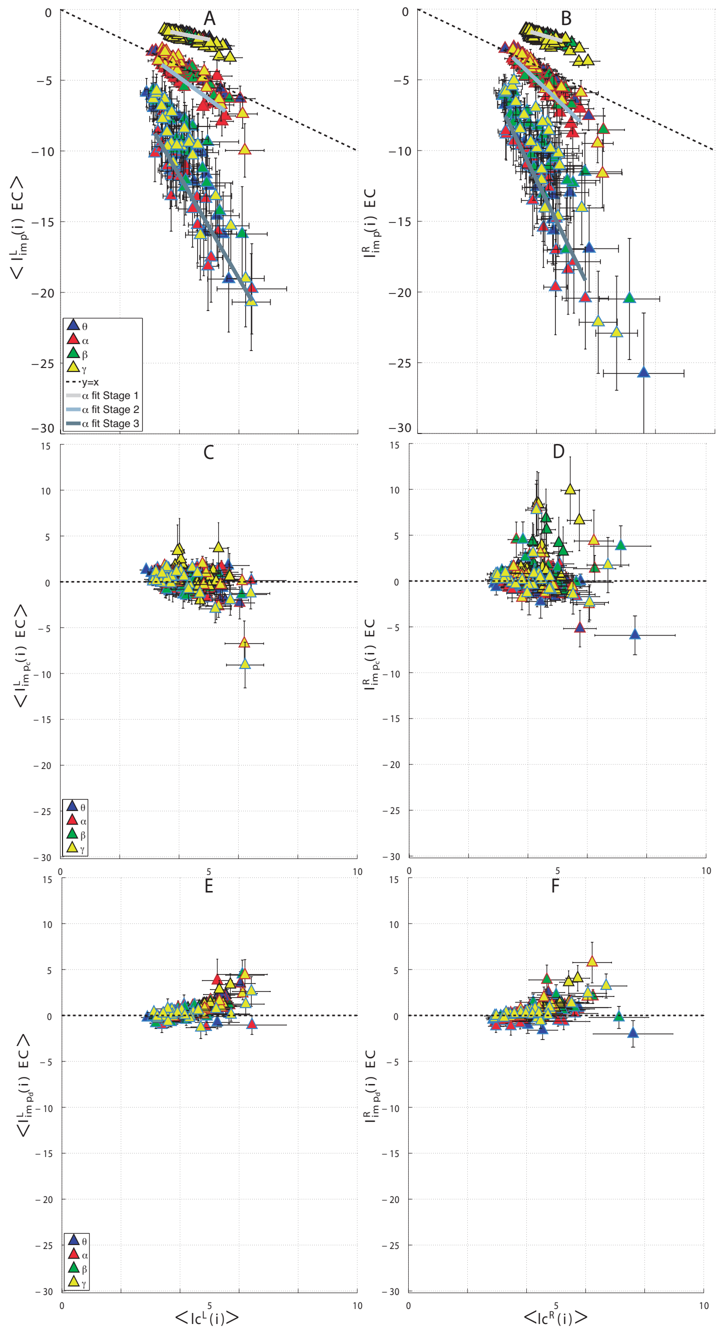

Figure S7: **Local impact for centrality, clustering and shortest path. EC condition**. Upper panels: Local impact on the hemispherical centrality for left (**A**), and right (**B**) hemispheres. Middle panels: Local impact on the clustering coefficient for the left (**C**) and right (**D**) hemispheres. Bottom panel: Local impact on the shortest path for left (**E**) and right (F) hemispheres.

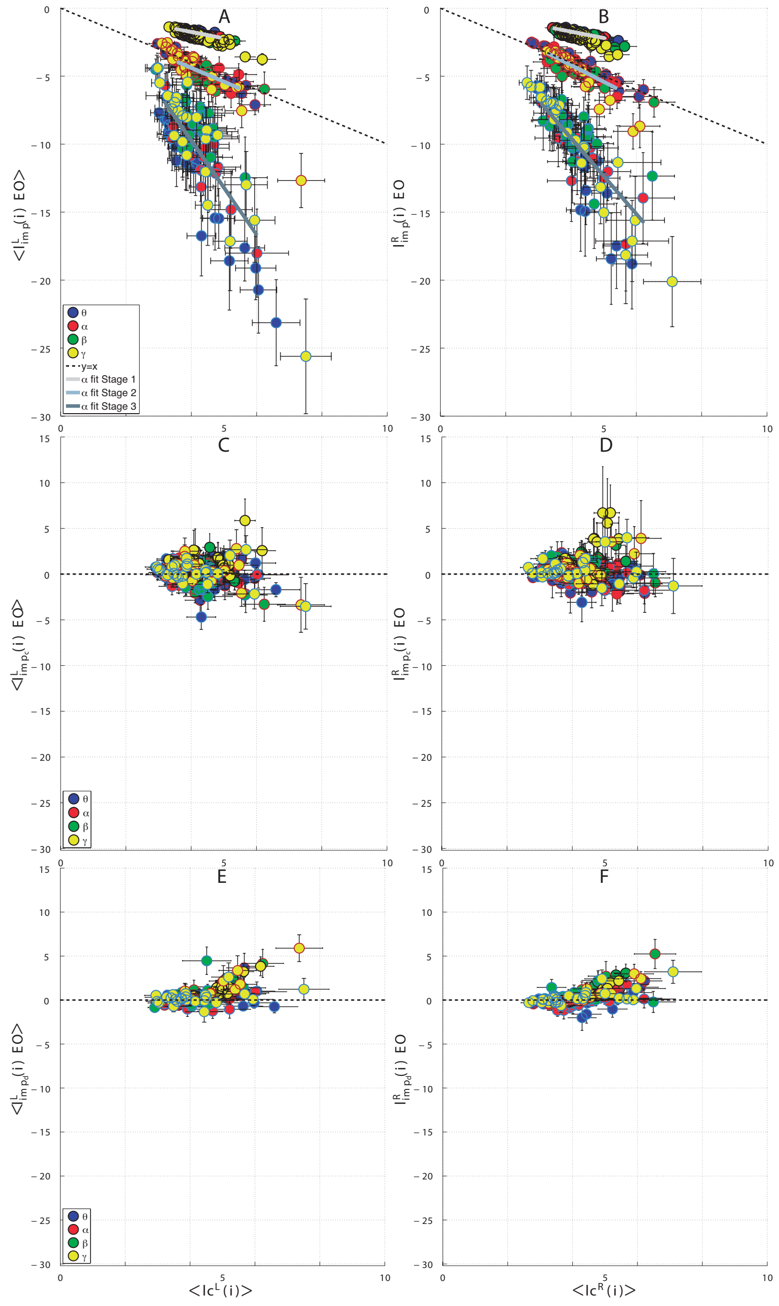

Figure S8: **Local impact for centrality, clustering and shortest path. EO condition**. Upper panels: Local impact on hemispherical centrality for the left (**A**), and right (**B**) hemispheres. Middle panels: Local impact on the clustering coefficient for the left (**C**) and right (**D**) hemispheres. Bottom panel: Local impact on the shortest path for the left (**E**) and right (F) hemispheres.
